# Resveratrol-Elicited PKC Inhibition Counteracts NOX-Mediated Endothelial to Mesenchymal Transition in Human Retinal Endothelial Cells Exposed to High Glucose

**DOI:** 10.3390/antiox10020224

**Published:** 2021-02-02

**Authors:** Roberta Giordo, Gheyath K. Nasrallah, Anna Maria Posadino, Francesco Galimi, Giampiero Capobianco, Ali Hussein Eid, Gianfranco Pintus

**Affiliations:** 1 Department of Medical Laboratory Sciences, College of Health Sciences and Sharjah Institute for Medical Research, University of Sharjah, Sharjah 27272, United Arab Emirates; robertagiordo2000@yahoo.it; 2 Department of Biomedical Sciences, College of Health Sciences member of QU Health, Qatar University, Doha 2713, Qatar; 3 Biomedical Research Center, Qatar University, Doha 2713, Qatar; 4 Department of Biomedical Sciences, University of Sassari, 07100 Sassari, Italy; posadino@uniss.it (A.M.P.); fgalimi@uniss.it (F.G.); 5 Gynecologic and Obstetric Clinic, Department of Medical, Surgical and Experimental Sciences, University of Sassari, 07100 Sassari, Italy; capobia@uniss.it; 6 Department of Basic Medical Sciences, College of Medicine, QU Health, Qatar University, Doha 2713, Qatar; 7 Biomedical and Pharmaceutical Research Unit, QU Health, Qatar University, Doha 2713, Qatar

**Keywords:** resveratrol, diabetes, fibrosis, NOX, oxidative stress, EndMT, retinopathy

## Abstract

Diabetes-associated long-term hyperglycaemia leads to oxidative stress-mediated fibrosis in different tissues and organs. Endothelial-to-mesenchymal-transition (EndMT) appears to play a role in diabetes-associated fibrotic conditions. Here, we investigate whether EndMT is implicated in the diabetic retinopathy fibrotic process and evaluate the possibility that resveratrol could counteract EndMT by inhibiting high glucose (HG)-induced increases in ROS. Primary Human Retinal Endothelial Cells (HRECs) were either pre-treated for 24 h with 1 µM resveratrol or left untreated, then glucose (30 mM) was applied at 3-day intervals for 10 days. qRT-PCR and ELISA were used to detect mRNA or protein expression of endothelial markers (CD31, CDH5, vWF) or mesenchymal markers (VIM, αSMA and collagen I), respectively. Intracellular ROS levels were measured with carboxy-DCFDA, while NOX-associated ROS levels were evaluated using the NADPH-specific redox biosensor p47-roGFP. Treatment of HRECs with HG increased intracellular ROS levels and promoted phenotype shifting towards EndMT, evidenced by decreased expression of endothelial markers concomitant with increased expression of mesenchymal ones. HG-induced EndMT appears to be mediated by NADPH-associated ROS generation as pre-treatment of HRECs with resveratrol or the NADPH inhibitor, diphenyleneiodonium chloride (DPI), attenuated ROS production and EndMT transition, suggesting that the effect of resveratrol on HG-induced ROS occurs via down-regulation of NADPH oxidase. It is worth noting that resveratrol or Chelerythrine, a Protein kinase C (PKC) inhibitor, reduce ROS and EndMT in HG-exposed cells, suggesting that NADPH activation occurs via a PKC-dependent mechanism. Taken together, our results provide the basis for a resveratrol-based potential protective therapy to prevent diabetic-associated complications.

## 1. Introduction

Diabetes is one of the major chronic diseases affecting the worldwide population [1]. Poor control of blood glucose establishes, over time, a state of chronic hyperglycemic condition in diabetic subjects [2]. Hyperglycemia-elicited long-term responses such as organ and tissue fibrosis appear to be essential players in the development of diabetes-associated vascular complications. These include retinopathy, nephropathy, neuropathy, cardiomyopathy as well as pathological conditions affecting the structure and function of both small and large blood vessels, leading to hypertension, peripheral vascular disease, cerebrovascular disorders and atherosclerosis [3,4,5,6].

Diabetic retinopathy (DR) is a common cause of vision impairment and blindness among the adult population [7]. DR is triggered by insults that lead to endothelial cell (EC) damage, which ultimately results in tissue remodeling and fibrosis [5]. Indeed, endothelial injury or dysfunction is often the first pathological event in many fibrotic diseases, thus highlighting the vital role of endothelium in vascular homeostasis [8]. The presence of activated fibroblasts or myofibroblasts, albeit of different origins, is a common feature of fibrotic tissues [9]. In this context, endothelial to mesenchymal transition (EndMT) represents one important source of mesenchymal cells [10]. During this process, ECs lose their characteristic cellular features or typical endothelial markers (e.g., VE cadherin, CD31, von Willebrand factor) and undergo a transition toward mesenchymal phenotype, significantly contributing to the ensuing fibrosis in the affected tissue [10,11]. Additionally, EndMT is marked by increased cell proliferation and migration, rearrangement of cell–cell junctions, as well as augmented secretion of extracellular matrix (ECM) proteins and expression of mesenchymal markers such as α-SMA, vimentin, and type 1 collagen [11,12]. 

Under hyperglycemic conditions, ECs failure to down-regulate glucose uptake, along with the consequent cell damage and phenotypic alteration, drives the mesenchymal fate of ECs [13]. In this regard, EndMT appears to be the predominant factor within diabetes-associated chronic complications such as diabetic nephropathy, cardiomyopathy and retinopathy [14,15,16,17,18]. Indeed, EndMT has been reported in glomerular endothelium of patients with diabetic nephropathy (DN), in high glucose-induced myocardial dysfunction, in the retina of diabetic mice with retinopathy and in the epiretinal membranes of patients with DR where it associates with high levels of Endotelin-1 [14,15,16,18,19]. 

Although the cellular and molecular determinants underpinning onset and progression diabetes-associated complication are still unclear, reactive oxygen species (ROS)-elicited vascular damage appears to play an important role. Indeed, ROS overproduction represents an upstream key event of different pathways involved in diabetic vascular damage [20,21]. Under physiological conditions, cellular antioxidant mechanisms keep levels of ROS in check [22]. However, aberrant regulation of these mechanisms leads to dramatic increases in ROS levels, thereby precipitating various pathological conditions [20,21,23]. Contextually, chronic hyperglycemia induces ROS and oxidative stress, modulates EC phenotypic changes and evokes vascular/microvascular remodeling [24,25]. Interestingly, both high glucose (HG) and ROS can amplify the expression of TGF-β, a well-known driver of EndMT in different tissues. In addition, up-regulation of TGF-β can, in turn, increase ROS generation by NADPH oxidase activation or through the mitochondrial electron transport chain [16,26,27]. As such, utilizing antioxidant agents to suppress excessive ROS production may hence be an effective approach to counteract HG-induced EndMT, as well as the associated fibrosis processes. Resveratrol, a polyphenol commonly found in many plants, has been widely reported to exert anti-oxidant, anti-apoptosis, anti-inflammatory and anti-angiogenic properties [28,29]. In this context, recent studies demonstrated that the anti-oxidant capacity of resveratrol is associated with anti-fibrotic potentials, suggesting that resveratrol can be a potential therapeutic agent to prevent and/or reverse diabetic-associated fibrotic conditions [30,31]. The present work was undertaken to further dissect the molecular mechanisms underpinning HG-induced EndMT and to investigate the ability of resveratrol to counteract HG-elicited effects on EC phenotype. For the first time, we show the involvement of Protein kinase C (PKC) in mediating NOX2 activation and ROS generation in response to HG, elucidating both the source of ROS and the associated signals. We also demonstrate the ability of resveratrol to counteract HG-induced EndMT in primary HRECs, highlighting its potential therapeutic value in the management of DR.

## 2. Materials and Methods

### 2.1. Cell Culture and Treatment

Human Retinal Endothelial Cells (HRECs) were purchased from ScienCell (Catalog number #6530). Cells were routinely grown in endothelial cell medium (ECM ScienCell #1001) with supplement (ScienCell ECGS #1052), 10% *v/v* fetal bovine serum and 1% 100 units/mL penicillin (Sigma-Aldrich, St. Louis, MO, USA), and 100 μg/mL streptomycin (Sigma-Aldrich) in 5% (*v*/*v*) CO_2_ humidified atmosphere at 37 °C [32]. HRECs were treated at 3-day intervals for 10 days with 5.5 mM D-Glucose (normal glucose, NG), 30 mM D-Mannitol (osmotic control, OC), 30 mM D-Glucose (high glucose HG), 1 µM resveratrol (1 day pre-treatment before addition of 30 mM Glucose), 5 mM N-acetyl cysteine (NAC) (1 h pretreatment before adding 30 mM Glucose), 2.5 µM Chelerythrine chloride (CHE) (1 h pretreatment before 30 mM Glucose) or 5 µM Diphenyleneiodonium chloride (DPI) (1 h pretreatment before 30 mM Glucose) [33,34,35,36]. Specifically, every 3 days, cells were divided into 2 aliquots; one aliquot of cells was used for some of the experiments (ROS, Viability, Apoptosis) and the other aliquot was cultured and stimulated again as indicated above. This procedure was repeated 3 times within 10 days. All experiments were performed in 2.5% fetal bovine serum. Resveratrol 10 mM stock solution was prepared in dimethyl sulfoxide (DMSO). The final DMSO concentration in the diluted treatment media was 0.01%. DMSO (0.01%) was used as a vehicle control. All reagents were purchased from Sigma-Aldrich (St. Louis, Missouri, United States).

### 2.2. Cell Apoptosis Assay

Cell apoptosis was assessed by using the fluorimetric kit APOPercentage (Biocolor Ltd., Carrickfergus, UK) following the protocol provided by the manufacturer. This assay has been employed with several adherent cell lines including endothelial cells [37,38] and uses a dye selectively imported by cells that are undergoing apoptosis. Necrotic cells cannot retain the dye and, therefore, are not stained. HREC cells were treated as previously described in the “cell culture and treatment” section and the apoptosis assay was performed at day 6 of treatment in 96-well black plates (BD Falcon). The APOPercentage dye 3,4,5,6,-tetrachloro-2′,4′,5′,7′-tetraiodofluorescein was added to each well (dilution 1:10) and cells were incubated for 30 more min at 37 °C in a cell incubator. After thoroughly washing, 100 µL of APOPercentage dye release reagent was added to each well, and the cell-bound dye recovered into solution was measured using a GENios plus microplate reader (Tecan) with excitation and emission of 530 and 580 nm, respectively. Results were calculated as the means ± SD of five measurements and expressed as a percentage of untreated control cells.

### 2.3. Cell Viability Assay

Cell viability was assessed in 96-well plates (BD Falcon) by using the colorimetric 3-(4,5-dimethylthiazol-2-yl)-2,5- diphenyltetrazolium bromide (MTT reagent) assay (Promega, Madison, WI, USA) [39,40]. Yellow MTT reagent enters the cells and passes into the mitochondria where mitochondrial dehydrogenases of viable cells cleave the tetrazolium ring, yielding reduced purple MTT formazan crystals, which are insoluble in aqueous solutions. This reduction occurs only when mitochondrial enzymes are active, and therefore conversion can be directly related to the number of viable cells. The formazan crystals can be dissolved in acidified isopropanol. The resulting purple solution is spectrophotometrically measured at 570 nm. An increase in cell number results in a large amount of MTT formazan formed and an increase in absorbance at 570 nm. After 24 h of RES treatment, 20 µL of MTT solution (2 mg/mL) in medium M199 were added to the cells and incubated at 37 °C in a cell culture incubator for 2 h. At the end of the incubation period, the solution was removed, and the purple formazan product was solubilized with acidic isopropanol (0.04 N HCl in absolute isopropanol). Then, plates were analyzed at 570 nm using a GENios plus micro-plate reader (Tecan). Results were calculated as the means ± SD of five measurements and expressed as a percentage of untreated control cells.

### 2.4. RNA Isolation and Quantitative Real-Time PCR

Total RNA was extracted from cells using Qiagen RNeasy purification kit (Cat # 74104), according to the manufacturer’s instructions with minor adjustments. For generating first strand cDNA, in each reaction, equal amounts of RNA were used as templates with SuperScript™ VILO™ cDNA Synthesis Kit (Thermo Fisher #11754). qPCR was performed on the QuantStudio™ 6 Flex Real-Time PCR System using TaqMan fast advanced master mix, custom TaqMan gene expression assays (AB Applied Biosystems) and approximately 20 ng of cDNA [41,42]. All reactions were performed in triplicate and data are showed as relative gene levels normalized to β-actin.

### 2.5. Measurement of Intracellular ROS

Intracellular ROS levels were determined using 6-carboxy-2′7′-dichlorodihydrofluorescein diacetate (Carboxy-H2DCFDA-Thermo Fisher). HRECs were incubated in 10 µM Carboxy-H2DCFDA for 30 min, and the fluorescence intensity was measured using GENios plus micro-plate reader (Tecan, Männedorf, CH) at an excitation wavelength of 485 nm and an emission wavelength of 535 nm. All fluorescence measurements were corrected for background fluorescence and protein concentration. Results were evaluated by comparison of five measurements and expressed as a means ± SD of the relative fluorescence unit (RFU) values [32,43].

### 2.6. Cloning of the p47-roGFP Biosensor Construct and Production of Lentiviral Particles

The coding sequence of human p47phox-roGFP2 was amplified by PCR from plasmid pcDNA3.1-p47-roGFP (kindly provided by Prof. George G. Rodney). Xba I and Sal I restriction sites (underlined in primers sequence) were added to the forward and reverse primers, respectively. (5′CGCTCTAGAATGGGGGACACCTTCATCCGT-3′), (5′AGTCGACACTTACTTGTACAGCTCGTCCATG-3′).

After purification, the PCR product was cloned into the lentiviral plasmid p156CMVGFP replacing the GFP gene at XbaI and Sal I sites. The final construct of p156CMV-p47roGFP was confirmed by sequencing. Lentiviral particles were prepared by transient transfection of the 293T/17 packaging cells as previously reported [44,45]. When cell confluence was about 70%, a mix of transgene expression plasmid, p156CMV-p47roGFP, and the third generation packaging plasmids (pVSVG, pREV, pMDL) was used to transfect the cells. Transfection was carried out using a calcium-phosphate solution consisting of a 1:1 mixture of 0.25 M of CaCl_2_:2X BBS (0.28 M NaCl, 0.05 M N,N-bis-(2-hydroxyethyl)-2-aminoethanesulfonic acid (BES), 1.5 mM Na_2_HPO_4_). The medium was exchanged 12 to 16 h post transfection and virus-containing media were harvested at 24 h intervals twice, starting 24 h after changing the medium. The collected medium was spun in a SW28 rotor for 2 h at 19,400 rpm in a L8-80M ultracentrifuge (Beckman); the pellet was re-suspended in 1 mL HBSS and spun again in the SW55 rotor for 2 h at 21,000 rpm in a L8-80M ultracentrifuge (Beckman). Finally, the second pellet was re-suspended in 200 μL in HBSS and the virus suspension was vortexed for 1 to 2 h at low speed before storing at −80 °C in 20 μL aliquots. Virus titer was determined by the p24 ELISA kit (PerkinElmer) according to the manufacturer.

### 2.7. Generation of HREC/p47roGFP Stable Cell Line

Generation of stable cell line was carried out as previously described [46,47]. Briefly, 50–60% confluent HREC were transduced using the optimized multiplicity of infection to reach an infection efficiency of nearly 95%. Cells were then incubated for 24 h, then an equal amount of fresh medium with no virus was added for an additional 24 h. At around 72–96 h, the efficiency was measured by flow cytometer analysis.

### 2.8. Determination of NOX2-Associated ROS Using NOX-Specific Redox Biosensor p47-roGFP

Determination of NOX2 dependent ROS production was investigated by using the NOX-specific redox biosensor p47-redox-sensing green fluorescent protein (p47-roGFP). P47-roGFP is a fusion of p47phox (a subunit of NADPH oxidase 2) and roGFP2, which has two fluorescence excitation maxima, 400 nm for the oxidized form and 485 nm for the reduced form. roGFP2 display rapid and reversible fluorescence changes in response to modification of redox potential. The ratios of fluorescence from excitation at 400 and 485 nm indicate the measure of oxidation and thus the redox state of the cell. HRECs transduced with p47-roGFP lentiviral particles were used for the specific indicted experiments. Fluorescence measurements of p47-roGFP-expressing cells were performed in black 96-well plates (Costar Inc, Corning, NY, USA) on a fluorescence microplate reader GENios plus (Tecan, Männedorf, CH). Results were evaluated by comparison of five measurements and expressed as a means ± SD of the relative fluorescence unit (RFU) values [41,46].

### 2.9. Protein Quantification by ELISA

Protein levels, of both endothelial and mesenchymal markers, were determined by ELISA kits (Elabscience cat. No: E-EL-H1640; E-EL-H1094; E-EL-H0977; E-EL-H2157; E-EL-H2168; E-EL-H0869), according to the manufacture’s protocol. Briefly, after treatment, cells were washed and then recovered in the protein extraction buffer. After normalizing samples for protein content, 100 µL of cell extract was incubated 90 min at 37 °C. In separate wells, a standard was also added. Following incubation, 100 µL of biotinylated detection Ab buffer was added and incubated for 1 h at 37 °C. Wells were then aspirated and washed 3 times with PBS buffer. Following incubation with 100 µL of HRP conjugate for 30 min at 37 °C, wells were aspirated and washed 5 times before adding 90 µL of substrate reagent. Substrate reagent was incubated for 15 min at 37 °C and the reaction was blocked with 50 µL of stop solution. Finally, the absorbance at 450 nm was measured and protein concentration was determined following the manufacture’s instruction [48,49].

### 2.10. Statistical Analysis 

Data are expressed as means ± S.D. of 5 different experiments. One-way analysis of variance (ANOVA) followed by a Newman–Keuls Multiple Comparison post-hoc test was used to detect differences of means among treatments, with significance defined as *p* < 0.05. Statistical analysis was performed using GraphPad Prism version 5.00 for Windows (GraphPad Software, San Diego, CA, USA).

## 3. Results and Discussion

### 3.1. High Glucose Induces EndMT in HRECs

Endothelial to mesenchymal transition (EndMT) is a process by which endothelial cells undergo deep phenotypic and physiologic alterations, which lead to a gradual loss of endothelial features and a concomitant acquiring of mesenchymal fates [10,11]. Endothelial dysfunction, one of the main factors in diabetic retinopathy and blindness progression, is due primarily to endothelial damage, which, if chronically persistent, can ultimately result in tissue remodelling and fibrosis [7]. Moreover, high glucose (HG) can damage blood vessels in the eyes even in the pre-diabetes state, when glucose levels are not high enough to be classified as diabetes [7]. A well-recognized method to investigate EndMT in tissues is determining the expression levels of endothelial and mesenchymal markers [9]. Endothelial markers are indeed partially downregulated in early EndMT and their expression progressively declines to a strong inhibition in late EndMT. On the contrary, mesenchymal markers are constantly upregulated from the early stage, although some of them, such as α-SMA and Col1, appear at the early stage, whereas others like Vim, MMP 2, MMP 9 and Col 3 are present from a later point [50,51].

**Figure 1:**
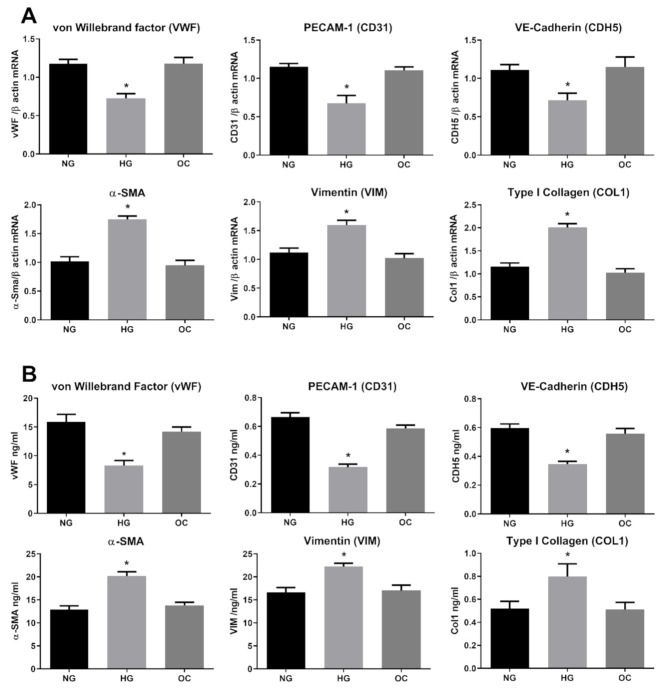
High Glucose induces endothelial-to-mesenchymal-transition (EndMT) in Human Retinal Endothelial Cells (HRECs). HREC cells treated with 5.5 mM glucose (normal glucose, NG), 30 mM glucose (high glucose, HG) or 30 mM D-mannitol (osmotic control, OC) for 10 days. mRNA levels of endothelial (vWF, CD31, CDH5) and mesenchymal markers (VIM, α-SMA, COL1) were determined by qRT-PCR using β-actin for normalization of data (**A**). Protein level (ng/mL) of endothelial (vWF, CD31, CDH5) and mesenchymal markers (VIM, α-SMA, COL1) were assessed by ELISA (**B**). Values are shown as mean ± SD, *n* = 5; *, significantly different from NG.

In order to determine the HG-exerted damage on HRECs, we first investigated the ability of HG to induce EndMT in our model. HRECs were exposed to a long and constant treatment (3-day intervals for 10 days) with 30 mM D-Glucose (high glucose; HG), 5.5 mM D-Glucose (normal glucose, NG), or 30 mM D-Mannitol (osmotic control, OC). As indicators of EndMT, three well established endothelial markers, CD31/PECAM, CDH5/Cadherin and vWF/von Willebrand factor, were employed, along with three well known mesenchymal markers, α-SMA, vimentin, and collagen I. As shown in Figure 1A, prolonged treatment with HG significantly modified the gene expression of all chosen markers, decreasing endothelial markers while upregulating the mesenchymal ones. The expression levels in OC treated cells were comparable with those in the control (NG), indicating that the observed phenomena were ascribable to the HG effect rather than an osmotic consequence (Figure 1). To further confirm the ability of HG to drive retinal endothelial cell phenotype toward mesenchymal features, we examined protein levels of the selected endothelial and mesenchymal markers. Indeed, ELISA results show that levels of cadherin, CD31 and vWF were all decreased, whereas those of α-SMA, Vimentin and Collagen I were all increased (Figure 1B). The current data confirm the ability of HG to induce the phenotypic switch in HRECs, supporting the hypothesis that myofibroblasts may originate from the ECs through the EndMT process in diabetic retina.

### 3.2. Resveratrol Counteracts HG-Induced EndMT

**Figure 2:**
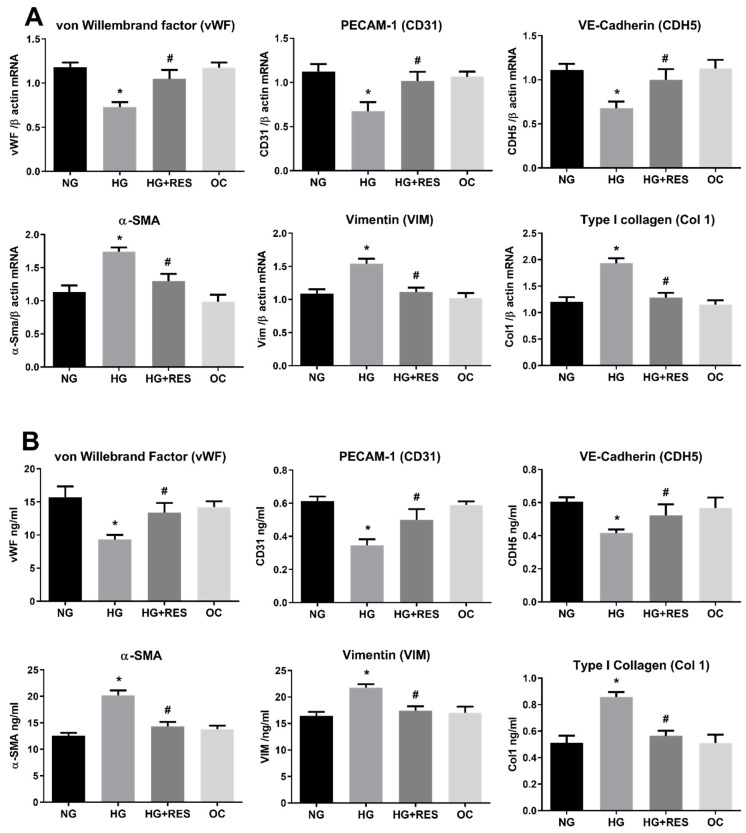
Resveratrol (RES) counteract HG-induced EndMT. HREC cells were pre-treated with 1 µM of RES for 24 h, before exposure to 5.5 mM glucose (normal glucose, NG), 30 mM glucose (high glucose, HG) or 30 mM D-mannitol (osmotic control, OC) for 10 days. mRNA levels of endothelial (vWF, CD31, CDH5) and mesenchymal markers (VIM, α-SMA, COL1) were determined by qRT-PCR using β-actin for normalization of data (**A**). Protein level (ng/mL) of endothelial (vWF, CD31, CDH5) and mesenchymal markers (VIM, α-SMA, COL1) were assessed by ELISA (**B**). Values are shown as mean ± SD, *n* = 5; *, significantly different from NG; #, significantly different from the HG.

Health benefits of resveratrol on eye diseases have been extensively reported. [52]. In diabetic rats, resveratrol significantly decreased hyperglycemia and oxidative markers, suppressing VEGF increase and vascular lesions associated with diabetic retinopathy [52,53]. In addition to its anti-fibrotic activity [54,55], recent studies showed that resveratrol suppresses epithelial–mesenchymal transition (EMT) through different pathways [31,56,57]. Moreover, resveratrol prevents hyperglycemia-induced EMT by virtue of its ability to inhibit the NADPH1 oxidase/ROS/ERK pathway, whereas inhibition of the TGFβ2/SMAD pathway is the primary mechanism involved in TGFβ-induced EMT counteraction [56,57]. EMT and EndMT are similar differentiation processes sharing some regulatory points including the critical role played by TGFβ [58,59,60,61]. However, EndMT is a less noted phenomenon and the molecular mechanisms underlying EndMT are largely unknown. To elucidate the effect of resveratrol on HG-induced EndMT, HRECs were pre-treated with 1 µM of resveratrol for 24 h, before adding 30 mM D-Glucose (high glucose HG), 5.5 mM D-Glucose (normal glucose, NG), or 30 mM D-Mannitol (osmotic control, OC). Both the culture medium and treatment compounds were replenished at 3-day intervals for the total duration of treatment (10 days). Since resveratrol can have potentially toxic effects, we chose 1 µM resveratrol, as this concentration does not affect ROS levels in HRECs [28,46,62]. As shown in Figure 2A, resveratrol was able to significantly abolish the HG-induced changes in the expression of marker genes for endothelial or mesenchymal phenotype (Figure 2B). Indeed, the effect of resveratrol on HG-induced protein modifications confirms its ability to suppress the mesenchymal fate of HRECs. Given the well-known antioxidant potential of resveratrol, the observed EndMT inhibition might be due to its antioxidant properties.

### 3.3. Resveratrol Counteracts HG-Induced ROS Generation and EndMT in HRECs

**Figure 3:**
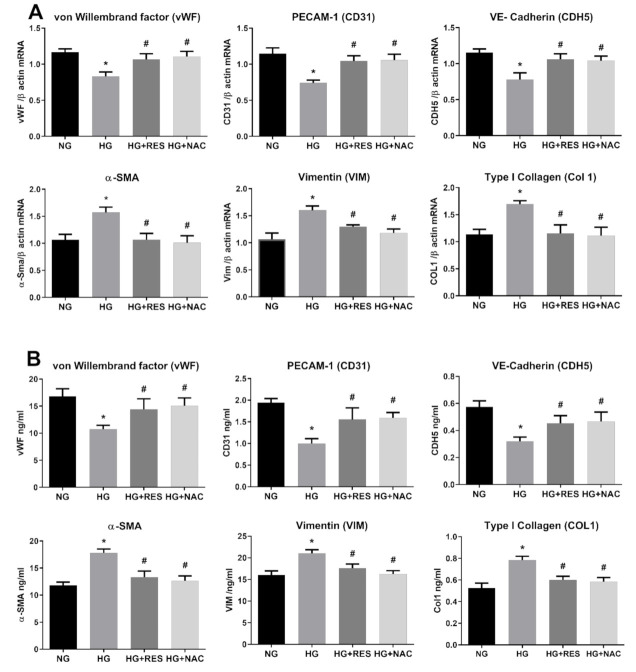
Antioxidant effect of N-acetyl cysteine (NAC) on HG-induced EndMT. HREC cells were pre-treated with 5 mM NAC for 1 h, before exposure to 5.5 mM glucose (normal glucose, NG), 30 mM glucose (high glucose, HG) or 30 mM D-mannitol (osmotic control, OC) for 10 days. mRNA levels of endothelial (vWF, CD31, CDH5) and mesenchymal markers (VIM, α-SMA, COL1) were determined by qRT-PCR using β-actin for normalization of data (**A**). Protein level (ng/mL) of endothelial (vWF, CD31, CDH5) and mesenchymal markers (VIM, α-SMA, COL1) were assessed by ELISA (**B**). Values are shown as mean ± SD, *n* = 5; *, significantly different from NG; #, significantly different from the HG.

**Figure 4:**
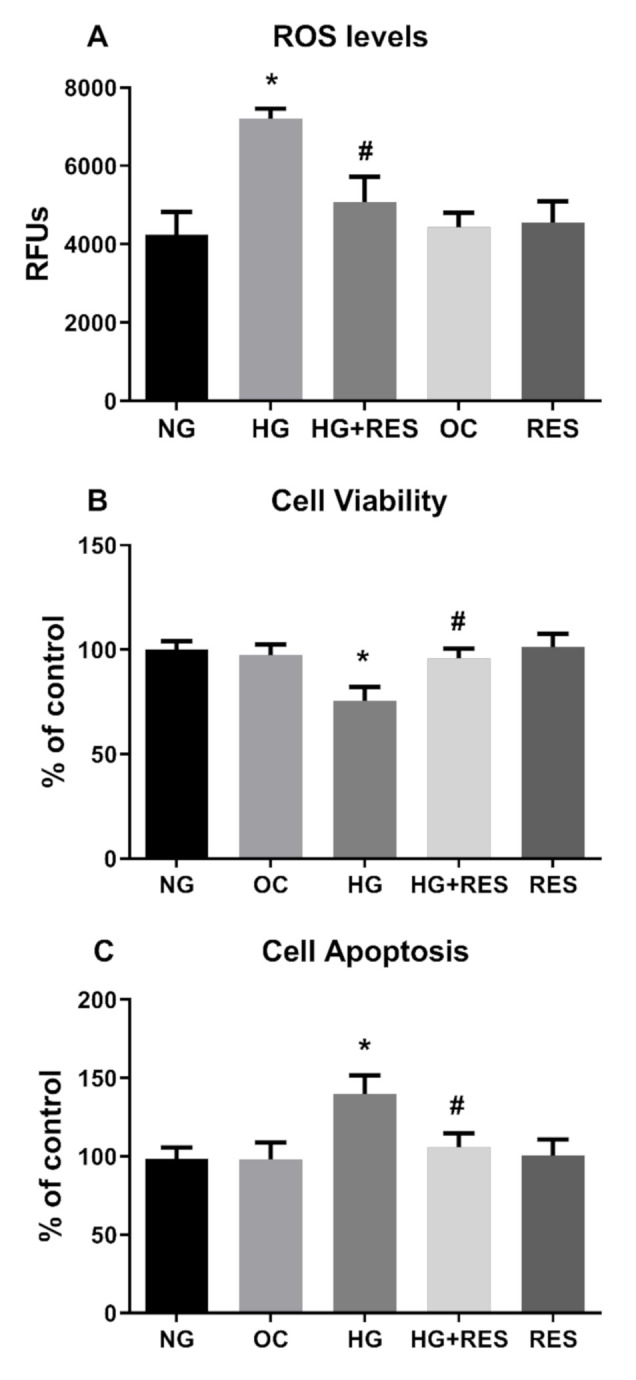
Effect of RES on HG-induced ROS production, cell viability and apoptosis. HREC cells were pre-treated with 1 µM of RES for 24 h before exposure to 5.5 mM glucose (normal glucose, NG), 30 mM glucose (high glucose, HG) or 30 mM D-mannitol (osmotic control, OC) for 10 days. ROS levels, cell viability and apoptosis were assessed every 3 days, as reported in the Materials and Methods section. Figures represent data obtained at 6 days. Values are expressed as mean ± SD (*n* = 5) of the relative fluorescence unit (RFU) (**A**). Results were calculated as the means ± SD (*n* = 5) and expressed as a percentage of untreated control cells (**B**,**C**); *, significantly different from NG; #, significantly different from HG.

Redox imbalance is well-documented to contribute to the pathogenesis of many diseases, such as cancer, cardiovascular disorders and diabetes [63,64,65]. Oxidative stress is a crucial player in the development of diabetes-associated cardiovascular complications, and hyperglycemia is the main cause of ROS accumulation and endothelial dysfunctions [65,66]. Recent studies show the ability of ROS to induce epithelial to mesenchymal transition or mediate endothelial cell conversion into fibroblasts [67,68]. In this regard, we speculated that ROS could be implicated in HG-induced EndMT in HRECs and be, at least in part, responsible for the DR associated fibrotic condition in vivo. To investigate this aspect, we tested the ability of N-acetyl cysteine (NAC), a well-known antioxidant compound [69], to counteract the HG-induced endothelial modifications observed in our DR in vitro vascular model. HRECs were pre-treated with 5 mM NAC for 1 h and successively exposed to 30 mM D-Glucose for 10 days. Protein and mRNA level analysis of the selected markers (Figure 3A,B) showed NAC’s ability to suppress HG-induced alterations. Indeed, NAC significantly abrogated HG-suppressed expression of endothelial markers (CDH5, CD3, vWF) while simultaneously inhibiting HG-induced expression of mesenchymal markers (Col 1, Vim and α-SMA) (Figure 3A,B). Figure 3 also reveals that the effect of resveratrol on HG-induced endothelial and mesenchymal markers modification is very similar to that of NAC, suggesting that the antioxidant property of resveratrol could be the underpinning mechanism of the observed effect. To confirm this assumption, HRECs cells were exposed to 30 mM glucose (HG) and 5.5 mM glucose (NG) for 10 days, with or without 1 µM resveratrol (1 day pre-treatment), and intracellular ROS were assessed at constant intervals (3 days) during the entire treatment. Our results show that HG was able to increase ROS generation, impair cell viability and induce apoptosis, confirming the close relationship between hyperglycemia and oxidative stress in DR (Figure 4). Moreover, resveratrol efficiently abolished HG-induced ROS production along with the associated phenomena, cementing our argument that it is the antioxidant capacity of resveratrol that suppresses hyperglycemia-associated ROS-mediated EndMT induction (Figure 4)

### 3.4. NADPH Oxidases Mediates HG-Induced ROS Generation

**Figure 5:**
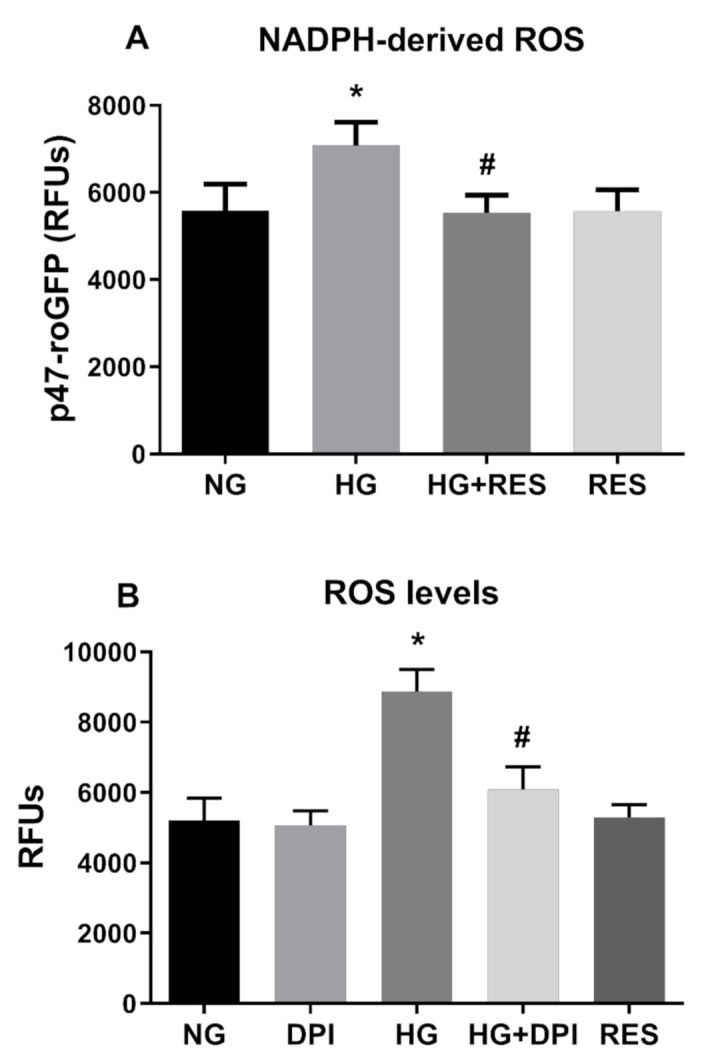
NADPH mediates ROS production induced by HG. RES efficiently counteract NADPH-dependent ROS formation induced by HG. HREC cells expressing p47-roGFP were pre-treated with 1 µM of RES for 24 h before exposure to 5.5 mM glucose (normal glucose, NG) and 30 mM glucose (high glucose, HG) for 10 days (**A**). Inhibitory effect of diphenyleneiodonium chloride (DPI) on NADPH-derived ROS. HREC cells were pre-treated with 5 µM of DPI for 1 h before exposure to NG and HG for 10 days. ROS levels and p47-roGFP activity were assessed every 3 days, as reported in the Materials and Methods section. Figures represent data obtained at 6 days (**B**). Values are expressed as mean ± SD (n = 5) of the relative fluorescence unit (RFU); *, significantly different from NG; #, significantly different from HG (**A**,**B**).

**Figure 6:**
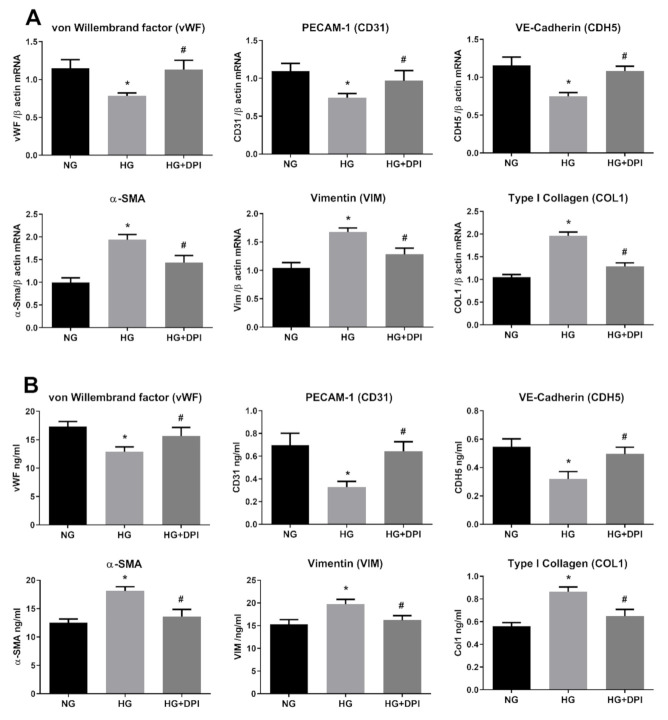
NADPH mediates HG-induced EndMT. HREC cells were pre-treated with 5 µM DPI for 1 h, before exposure to 5.5 mM glucose (normal glucose, NG) and 30 mM glucose (high glucose, HG) for 10 days. mRNA levels of endothelial (vWF, CD31, CDH5) and mesenchymal markers (VIM, α-SMA, COL1) were determined by qRT-PCR using β-actin for normalization of data (**A**). Protein level (ng/mL) of endothelial (vWF, CD31, CDH5) and mesenchymal markers (VIM, α-SMA, COL1) were assessed by ELISA (**B**). Values are shown as mean ± SD, *n* = 5; *, significantly different from NG; #, significantly different from the HG.

Although our data confirmed the involvement of oxidative stress in HG-induced EndMT, the source of ROS associated with this phenomenon remains unclear. During diabetes-associated complications, ROS can be generated by different pathways and enzymes, although NADPH oxidases of the NOX family seem to be the main source [70]. Recent studies, conducted both in cultured retinal endothelial cells and in animal models, highlight a direct involvement of NOX2- and NOX4-generated ROS in diabetic retinopathy [71]. Activation of the NOX enzyme complex requires translocation of the cytosolic component (p47phox) to the plasma membrane, where its association to NOX2 evokes ROS production. To specifically assess NOX activity, we generated a stable cell line by transducing HRECs with lentiviral particles carrying the p47-roGFP [47]. This chimeric protein has been obtained by fusion of the redox sensitive biosensor (roGFP) with the NOX organizer protein p47phox and has been reported to be an efficient tool for assessing NOX2 activation and, in turn, ROS generation by this specific NOX [72,73]. As displayed in Figure 5A, stimulation of p47-roGFP-expresssing HRECs with 30 mM glucose resulted in increased NOX activity, a phenomenon significantly abrogated by the presence of resveratrol. The current data incriminate NOX as part of the signaling machinery underpinning HG-induced ROS generation and suggest that resveratrol may suppress EndMT via the inhibition of this ROS generating enzyme. To confirm NOX as the primary source of hyperglycemia associated responses in HERCs, we tested whether DPI, a NADPH inhibitor, diminishes both HG-induced ROS or EndMT transition. To this end, HERCs were pre-treated for 1 h with DPI, before the addition of 30 mM Glu, and the treatment was repeated every three days for ten days. During the experimental time-course, ROS were assessed every three days before repeating the treatment. As shown in Figure 5B, HG-induced ROS production was significantly attenuated by DPI. In addition, pre-treatment with DPI also negated the downregulation of endothelial markers (CD31, CDH5, vWF), (Figure 6A) simultaneously reducing the expression of mesenchymal markers (α-SMA, Vim, Col1), both at the mRNA and protein levels (Figure 6A,B). Interestingly, as shown in Figure 5 and Figure 6, the effects of DPI on both ROS production and EndMT are very similar to those of resveratrol. The current data confirm the main role of NADPH oxidase in diabetes-associated fibrotic responses and indicate its inhibition as the primary underpinning mechanism of resveratrol inhibition upon HG-induced EndMT.

### 3.5. HG Activates NADPH Via PKC

**Figure 7:**
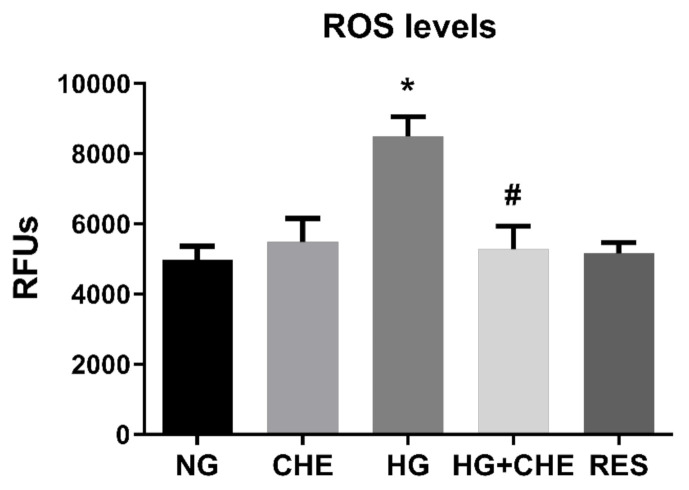
Effect of CHE on HG-induced ROS production. Inhibitory effect of Chelerythrine chloride (CHE) on PKC activity induces a decreasing of HG-induced ROS production. HREC cells were pre-treated with 2.5 µM CHE for 1 h before exposure to 5.5 mM glucose (normal glucose, NG) and 30 mM glucose (high glucose, HG) for 10 days. ROS levels were assessed every 3 days, as reported in the Materials and Methods section. Figures represent data obtained at 6 days. Values are expressed as mean ± SD (*n* = 5) of the relative fluorescence unit (RFU); *, significantly different from NG; #, significantly different from HG.

**Figure 8:**
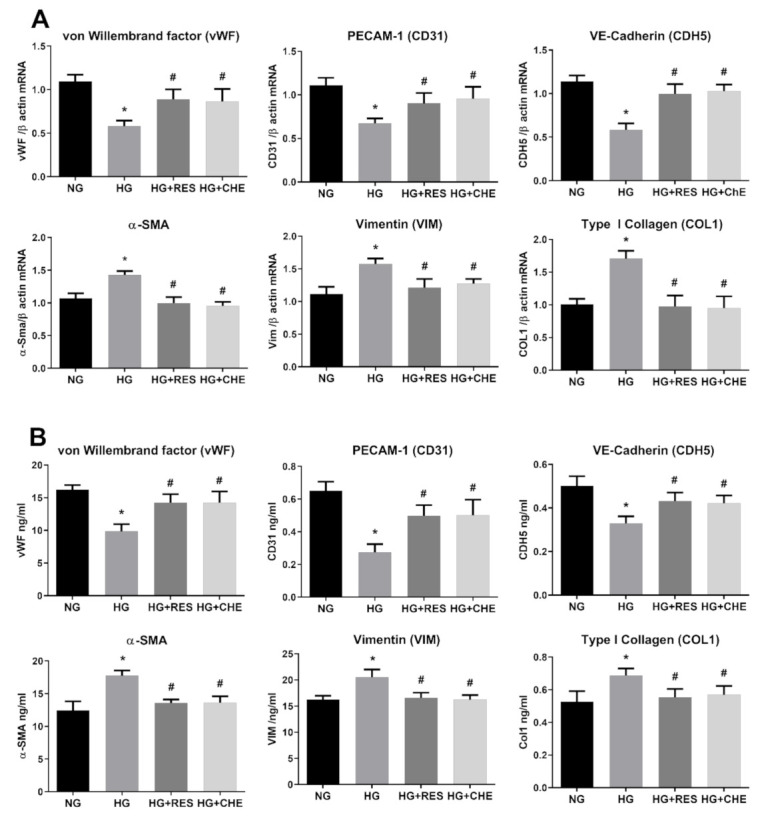
HG activates NADPH via PKC. HREC cells were pre-treated with 2.5 µM CHE for 1 h, before exposure to 5.5 mM glucose (normal glucose, NG) and 30 mM glucose (high glucose, HG) for 10 days. mRNA levels of endothelial (vWF, CD31, CDH5) and mesenchymal markers (VIM, α-SMA, COL1) were determined by qRT-PCR using β-actin for normalization of data (**A**). Protein level (ng/mL) of endothelial (vWF, CD31, CDH5) and mesenchymal markers (VIM, α-SMA, COL1) were assessed by ELISA (**B**). Values are shown as mean ± SD, *n* = 5; *, significantly different from NG; #, significantly different from the HG.

We next wished to further dissect the molecular mechanisms underpinning the inhibitory effect of resveratrol on HG-induced ROS increase and EndMT activation. In this regard, a potential role of protein kinase B (Akt), Raf kinase inhibitor protein (RKIP), and miRNA-containing exosomes in diabetes-induced EndMT has been postulated [74,75,76]. Nonetheless, the molecular machinery linking diabetes-associated oxidative stress and EndMT is still missing. PKC activation is one of the HG-induced primary mechanisms leading to endothelial dysfunction in diabetes [77,78,79]. Hyperglycemia and diabetic conditions have been reported to increase diacylglycerol (DAG) concentration, which, in turn, activates PKC that increases NADPH oxidase activity, thus promoting ROS generation [80,81]. We hypothesized that resveratrol acts upstream of NADPH blocking NOX-mediated ROS generation by direct inhibition of PKC. To this end, we evaluated the effect of PKC inhibitor Chelerythrine (CHE) on both HG-indued ROS production and EndMT activation. To this end, HRECs were pre-treated for 1 h with 2.5 µM CHE before the addition of 30 mM Glucose. HG treatment was carried on for 10 days and intracellular ROS were measured at constant intervals (3 days). As depicted in Figure 7, HG-induced ROS was significantly reduced by CHE, confirming a role for PKC in activating NOXs. Interestingly, CHE was also able to counteract EndMT induced by HG. Indeed, both gene and protein expression levels of selected endothelial (CD31, CDH5, vWF) and mesenchymal (Col1, α-SMA, Vim) markers did not significantly differ from the control (Figure 8A,B). Together, these results support the notion that PKC plays a key role in HG-induced ROS production and EndMT activation. Since the effects of CHE on both HG-indued ROS and EndMT are very similar to those of resveratrol, we hypothesize that the observed effects of resveratrol on both NOX inhibition and EndMT are mediated by PKC. Indeed, this would not be surprising as we recently showed a direct interaction of resveratrol with the PKC [39].

## 4. Conclusions

High glucose blood levels lead to long term diabetes-associated fibrotic complications including diabetic retinopathy. Myofibroblasts originating from tissue resident endothelial cells appear to be the primary source of diabetes-associated fibrotic conditions including DR. Here, we confirmed that HG promotes the phenotype switch of HRECs, and suggest that myofibroblasts in the diabetic retina may originate from ECs through the EndMT process. HG-induced EndMT appears to be mediated by NADPH-associated ROS generation as ECs pretreatment with a NOX inhibitor was able to effectively abolish it. Moreover, both the antioxidants resveratrol and NAC were able to significantly prevent HG-induced oxidative stress and ECs phenotype modification, suggesting a primary role for oxidative stress in triggering the DR-associated fibrotic process. Further experiments indicated PKC positioned upstream both the HG-elicited ROS increased and EndMT activation, as well as mediating the resveratrol-suppressed effects. Our findings show that PKC inhibition reduces both ROS and EndMT in cells exposed to high glucose, suggesting that NADPH activation and EndEM occur via PKC, which also appears to be key mediator of resveratrol in these cells. We therefore reasonably postulate that activation of the DAG-PKC-NADPH pathway is responsible for EndMT induction in retinal endothelial cells exposed to HG. Due to improved diabetes care and diabetes self-management, glucose levels are moderately elevated in most diabetic patients [82]. However, in in vivo experimental models of poorly controlled diabetes, glucose levels above 20–30 mmol/L have been reported and vascular dysfunction develops over weeks or a few months [82,83]. In vivo evidence also suggests that, after chronic consumption, resveratrol, at a concentration of 1.7 µM, is detectable in plasma up to 1 week after wash-out [84]. The above observations indicate that the concentrations employed in our work could be representative of an in vivo physiologically relevant scenario. Taken together, our findings indicate EndMT as an important mechanism in DR development and suggest the use of antioxidants as a potential therapeutic approach to prevent the progression of diabetic-associated complications including fibrosis.

## Data Availability

All data is contained within the article.
